# A cross-sectional exploratory study of knowledge, attitudes, and practices of emergency health care providers in the assessment of child maltreatment in Maputo, Mozambique

**DOI:** 10.1186/s12873-018-0162-9

**Published:** 2018-05-09

**Authors:** Liliana Pinto, Adriana Lein, Raquel Mahoque, David W. Wright, Scott M. Sasser, Catherine A. Staton

**Affiliations:** 1 Ordem dos Médicos de Moçambique (Mozambique Medical Council), Maputo, Mozambique; 2 Duke Global Health Institute, Duke University, 310 Trent Drive, Durham, North Carolina 27710 USA; 3 World Health Organization, Rua Pereira Marinho 280, CP 377 Maputo, Mozambique; 4 Emory Department of Emergency Medicine, 531 Asbury Circle, Annex Building, Suite N340, Atlanta, GA 30322 USA; 5 Department of Emergency Medicine, Greenville Health System, University of South Carolina School of Medicine Greenville, Greenville, SC 29605 USA; 6 Division of Emergency Medicine, Department of Surgery, Duke University Medical Center, 2301 Erwin Road, Durham, North Carolina 27710 USA; 7 Division of Global Neurosurgery and Neurology, Department of Neurosurgery, Duke University Medical Center, 2301 Erwin Road, Durham, North Carolina 27710 USA

**Keywords:** Child maltreatment, Emergency care services, Health care providers, Mozambique-Africa, Human rights abuses

## Abstract

**Background:**

In Mozambique, and other low-income countries (LICs), there is little information on the burden of child maltreatment (CM). Emergency care services (ECS) play an important role in recognizing, treating, and intervening in situations of CM. We aim to identify knowledge, attitudes, and practices regarding CM among health care providers in ECS at Mavalane General Hospital in Maputo, Mozambique.

**Methods:**

This exploratory cross-sectional study evaluates the knowledge, attitudes, and practices of health care providers to diagnose and treat cases of CM. A 25 min, pilot-tested verbal interview questionnaire was administered to 49 physicians and nurses working in ECS at Mavalane General Hospital. Interviews were completed between October–November 2010. Data were managed and analyzed in SPSS 14.0 and descriptive statistics were generated.

**Results:**

Of 49 health care providers, 83.6% reporting receiving no, or very little CM education or training. Only 61.2% had knowledge of physical abuse as a main form of child maltreatment and 38.8% were able to identify corresponding symptoms of physical abuse. Sexual abuse as a main form of CM was mentioned by 26.5 and 2% cited its symptoms. While 87.7% of health care providers strongly agreed or agreed that they hold an important role in preventing CM, 51.1% also strongly disagreed or disagreed that they feel confident diagnosing and treating CM cases. In regards to follow-up, 14.3% strongly disagreed or disagreed that they know where to refer victims for further follow-up and an additional 14.3% did not know whether they agreed or disagreed.

**Conclusion:**

This study revealed knowledge gaps in emergency health care provider knowledge of the main forms of CM and their symptoms. The fact that a majority of health care providers in our sample did not receive information specific to CM in their medical education and training could explain this gap, as well as their unawareness of where to refer victims. Given that health care providers believe they play an important role in identifying and treating CM, future research should focus on raising physician awareness of CM and developing education and training materials grounded in cultural contexts to build response capacity in Mozambique and other LICs.

## Background

Child maltreatment (CM), the physical, sexual and emotional abuse, neglect, or exploitation of under 18 populations carries a heavy social cost and is a serious human rights and public health issue [1–3]. Distinct from other forms of violence against children, child maltreatment CM refers specifically to acts perpetrated by parents or caregivers [4]. CM is alarmingly pervasive; globally, an estimated 60% of children aged 2–14 experience frequent physical punishment and as many as 70% are victims of psychological abuse from their caregivers [5]. Rates of CM are the highest in low-income countries, particularly in the African region where the heaviest burden is observed [5–7]. Studies from Africa have found that between 75 and 94.4% of children experienced physical abuse from caregivers in the past year and 58.3–64% experienced emotional abuse [6, 8, 9]. The highest prevalence of child sexual abuse is also seen in Africa, with 34.4% of children experiencing a form before the age of 18 [10]. CM causes a range of long-term physical and mental sequelae, including neurological, cognitive, affective, and social disorders [11, 12], and is associated with poor school performance [13] and internalized acceptance towards gender-based violent attitudes in girls [14].

CM is closely tied to the social and cultural context. Numerous studies from Africa point towards poor knowledge of what constitutes abuse and accepting attitudes of abusive behaviors in cultures where corporal punishment and other forms of harsh discipline are entrenched in traditions and commonly practiced as main drivers [15–18].CM is also associated with poverty and socioeconomic status [8, 19, 20] and exposure to other forms of violence, namely domestic violence, within the household [14, 21]. Due to the normalization of CM, cases are rarely reported and it’s often termed a silent phenomenon [4, 22–25]. Literature from high-income countries suggests that training health care providers to be aware of abusive behaviors, recognize warning signs, and refer suspected cases to appropriate services might be one effective form of intervention and prevention [26–29]. This is due to the fact emergency departments are often critical entry points into crisis response systems [30]. Despite the potential for emergency care services (ECS) to act as a first line of defense against CM, studies of this population from Sub-Saharan Africa are limited.

Existing studies from the region related to CM management in a hospital tend to focus on the profile of injuries, rather than health care providers. One study of physicians in the region found poor knowledge in correctly diagnosing cases of CM [31]. Yet, studies from ECS in Africa have found the burden of intentional injuries to be higher, between 18 and 21.3% [32, 33]. Training health care providers to recognize warning signs of CM is critical for timely intervention and prevention [27, 34]. Given the cultural context, knowledge of symptoms of CM alone may be insufficient. Thus, there is a need for reliable studies from Africa that take a holistic approach to examining barriers to diagnosis and intervention in suspected CM, which may include limited knowledge [31, 34], negative attitudes towards their role to intervene [23], and uncertainty in diagnosing and confusion about their responsibility to report cases [35, 36]. Consequently, there is a need for reliable studies to better understand current deficiencies and their determinants in the diagnosis and case management of child maltreatment victims in emergency departments.

In Mozambique, a low-income country in Southeast Africa, 5696 cases of violence against children were reported to police authorities and 190,000 cases were reported to the Child Help Line (*A Linha Fala Criança*) in 2015 alone [33].The actual prevalence of CM is likely underestimated due to fragmented surveillance, limited legal infrastructure, and the absence of children’s rights protections [22]. While the Ministry of Health has recently adopted a new approach centered on education and preparedness, the capacity to identify and treat CM in emergency care services in Mozambique (ECS) is still unknown. This study aims to advance current understandings of ECS providers’ knowledge, attitudes, and practices in identifying and intervening in situations of CM. Findings will assist in highlighting key areas to focus policies and trainings to support response capacity for this first line of defense.

## Methods

### Study design and ethical approval

This is an exploratory cross-sectional study of knowledge, attitudes, and practices of health care providers working in ECS in relation to cases of CM at Mavalane General Hospital (MGH). Ethical approval for this study was obtained from Mozambique’s National Committee for Bioethics for Health (*Comité Nacional de Bioética para Saúde*).

### Setting

This study was carried out at MGH located in the federal capital of Maputo. MGH is a tertiary care facility with a catchment area of 19 neighborhoods, a covered population of 521,333 inhabitants (47.6% of the population of Maputo). MGH is the only primary hospital in its region and offers 24-h ECS, contracting approximately 40 physicians and 20 nurses. Hospital infrastructure for ECS consists of three triage rooms, each staffed by three general physicians and organized around three shifts of morning, afternoon, and night. Patients are first triaged prior to receiving treatment.

### Participants

Sixty health care providers working in ECS at MGH were selected through a purposive sample and invited to participate in this study. Under time and resource constraints, this number was considered sufficient for data collection. Study eligibility was based on 1) status as a current clinical health care provider in ECS at MGH, and 2) voluntary agreement and written informed consent. Participants were also informed that they could withdraw participation or choose not to respond to questions at any point in the study without repercussions. Of the 60 health care providers invited to participate in the study, 49 consented to participate (82%).

### Data collection

This study relied on primary data collected through direct interviews with participants by interviewers trained in administration by the study’s principal investigator. Interviews occurred in the emergency department of MGH at the time and location preferred by participants to not interfere with work schedules and maximize privacy. Data collection between October and November 2010.

### Survey

The data collection instrument for this study was a survey created through a content analysis of a similar study conducted by the United Nations Children’s Fund (UNICEF) in Nepal [23]. The original study contained open-ended questions related to health care providers’ knowledge, attitudes, and practices in the assessment of and response to CM. Qualitative data were systematized and analyzed through to facilitate the classification and development of items to measure the three thematic categories of knowledge, attitudes, and practices.

Prior to the initiation of the study, a pilot test was carried out at José Macamo General Hospital, also in Maputo. This hospital was selected for its similar location and capacity to MGH and adjustments were made to the survey following the pilot to account for the context of Mozambique.

Our survey consisted of a combination of demographic, multiple-choice (E.g. “Identify which of the below are forms of child maltreatment”) yes-no (E.g. “Does child maltreatment cause long-term adverse effects on child development?”), and Likert-type scale items (E.g. “Indicate your level of agreement with the statement that ‘health care providers have an important role in treating CM”, organized in the following four sections: 1) sociodemographic characteristics, 2) health care provider knowledge of CM, 3) health care provider attitudes of CM, and 4) health care provider practices in recognizing and responding to CM. In total, there were seven demographic questions, 18 questions to measure knowledge, 20 questions to measure attitudes, and eight questions to measure practices.

Surveys, containing both qualitative short answer as well as quantitative Likert scale questions, were administered by an interviewer trained by the study’s principal investigator. Interviews were conducted individually, maintaining confidentiality and anonymity of responses by storing data by a study number only without participants’ identifying information.

### Data analysis

Following data collection, previously coded surveys were reviewed to verify and validate responses. Descriptive statistics (frequencies and percentages) were used to report and characterize responses. Responses to the demographic, multiple choice, and Likert-type scale items were included in our analysis.

## Results

Of the 60 health professionals identified as eligible, 49 (81.6%) consented to participate and were interviewed. Physicians made up 60% of health professionals interviewed. The final sample was composed primarily of women (69.4%). The mean respondent age was 32 years and most respondents were between 25 and 45 years (81.6%). Generalist physicians made up 57.1% of respondents. Table 1 details the sociodemographic characteristics and professional classifications of study participants.

**Table 1:** Sociodemographic characteristics and professional classifications of study participants

Sociodemographic characteristics	Frequency (n)	Percent (%)
Sex		
Male	15	30.6
Female	34	69.4
Age		
˂25 years	7	14.3
25–45 years	40	81.6
> 45 years	2	4.1
Professional classifications		
Generalist physician	28	57.1
Nurse	19	38.7
Pediatrician	1	2.0
Surgeon	1	2.0

Data on previous education and training specific to CM was also collected to characterize health care providers’ background (Table 2). On identifying or responding to CM, 83.6% of respondents disclosed having received no education/training or almost no education/training. Only 14.3% of respondents indicated having received some education/training specific to CM. Regarding knowledge, health care providers interviewed were asked to cite the main forms of CM (Table 3); 61.2% of respondents cited physical violence/aggression as being a main form of CM, 26.5% cited sexual abuse, and 12.2% cited emotional/psychological abuse. Although the majority of respondents reported physical violence/aggression as the main form of CM, 8.2% of respondents did not identify any of the forms of CM.

**Table 2:** Level of education and training specific to child maltreatment

Category	Frequency (n)	Percent (%)
No education nor training	35	71.4
Almost no education nor training	6	12.2
Some education and training	7	14.3
Does not know	1	2.0

**Table 3:** Knowledge of the main forms of child maltreatment^a^

Category	Frequency (n)	Percent (%)
Physical violence/aggression	30	61.2
Sexual abuse	13	26.5
Negligence	5	10.2
Exploitation	5	10.2
Emotional/psychological abuse	6	12.2
Others	11	22.4
Does not know	4	8.2

^a^Participants were asked to identify all responses that applied and responses are not mutually exclusive

Knowledge of health care providers to recognize CM was also measured based on awareness of typical physical symptoms. Approximately 38.8% of the health care providers surveyed identified scarring and bruising at different stages of healing, 32.6% identified marks on the body in the form of objects such as a belt or cigarette burn, 4.1% identified burns, and 2.0% identified the presence of a sexually transmitted infection. Additional symptoms were mentioned by 20.4% of respondents; including malnutrition, scratches, ecchymosis, abrasions, and redness of the lips, lacerations in the case of sexual abuse, swelling, fear, ulceration, and depression. It is important to note that 8.2% of health care providers revealed that they did not know the typical physical symptoms of CM.

In relation to attitudes, 87.7% of respondents strongly agreed or agreed that health care providers hold an important role in the prevention of CM. Only 8.2% disagreed that health care providers hold an important role in the prevention of CM. Respondents were also asked their level of agreement with the attitude that “victim follow-up is not required because it is not important.” A majority of respondents (86.2%) strongly disagreed or disagreed with that statement; only 10.2% strongly agreed or agreed. In contrast, health care providers expressed low confidence diagnosing and treating CM (Table 4). Over half (51.1%) strongly disagreed or disagreed that they felt confident diagnosing and treating CM.

**Table 4:** “I feel confident diagnosing and treating cases of child maltreatment”

Category	Frequency (n)	Percent (%)
Strongly disagree	3	6.2
Disagree	22	44.9
Neutral	4	8.2
Agree	15	30.6
Strongly agree	1	2.0
Does not know	3	6.1
Did not respond	1	2.0

To examine practices, participants were asked about the existence of a standard protocol for the care of victims of CM. Just 55.1% of respondents correctly stated that there is not a care protocol for cases of CM, while 24.5% incorrectly referred to an existing care protocol, and 16.3% did not know if such a protocol is in place. Respondents were then asked if they know where to refer for further treatment (Table 5). Most respondents (69.3%) strongly agreed or agreed that they know where to refer victims; 14.3% strongly disagreed or disagreed and 14.3% of respondents admitted that they do not know.

**Table 5:** “I know where to refer cases of child maltreatment for further follow-up”

Category	Frequency (n)	Percent (%)
Strongly disagree	2	4.1
Disagree	5	10.2
Neutral	0	0
Agree	32	65.3
Strongly agree	2	4.1
Does not know	7	14.3
Did not respond	1	2.0

## Discussion

Our study is one of the first evaluations in a LIC of the knowledge and practice deficiencies in diagnosing and treating CM in an emergency department. Health care providers in ECS are the front line of the health system and can reduce harm by identifying CM when and before it happens. This study adds much needed literature on Sub-Saharan Africa, where rates are among the highest in the world [5–7]. The latest global guidelines for the prevention of CM emphasize the vital role of the health sectors of LMICs and our study offers evidence to guide the creation of new policies and investment in human resources in this context [25]. We found a significant gap in health care providers’ knowledge of the forms of CM and their symptoms beyond physical violence. The attitudes expressed by health care providers overwhelmingly suggest agreement with their role to intervene. However, our findings reveal a disconnect between the attitudes held by health care providers and the training they receive; one plausible explanation for this discrepancy is a lack of consensus and blurred boundaries around what constitutes CM amid a context in which the harsh punishment of children in normalized and widespread.

A slight majority (61.2%) of participants identified physical violence as a main form of CM. Yet, only 26.5% cited sexual violence and even fewer (12.2 and 10.2%, respectively) referred to psychological abuse and exploitation. Conformingly, bruises/scarring and marks on the body were the most frequently mentioned symptoms, and responses pointing to symptoms of sexual abuse were virtually absent. These findings of health care providers having knowledge predominantly of physical abuse are consistent with those of CM studies in international literature [37, 38]. A central challenge in the correct diagnosis of CM is that fact that most pediatric injuries globally (90%) are in fact unintentional and occur without malice [39]. Accordingly, future interventions to educate health care providers and increase knowledge should focus on individual-level risk factors for CM and encourage consideration of patient history and demographics in the assessment. Moreover, there is a need to better understand and treat negligence and emotional abuse as forms of CM, as well as provide long-term support to victims [26, 37, 38, 40].

Despite a limited knowledge base, the attitudes expressed in our study show strong agreement that health care providers have an important role in preventing CM, as 87.7% of respondents agreed. Similar attitudes regarding a responsibility to document, report, and treat suspected CM have been affirmed in some previous studies of diverse health care providers [37, 41]. Likewise, attitudes indicated a strong commitment to appropriately managing cases of CM, with an overwhelming majority of participants disagreeing that victim follow-up is not important. At the same time, over half of our participants did not feel confident in diagnosing and treating CM; only 2.1% strongly agreed that they felt confident. This is not surprising as 71.4% of participants did not receive any education or training specific to CM. Health care providers who have received training specific to CM have been found to have significantly greater knowledge of reporting procedures, compared to physicians who did not receive training specific to CM [27]. Although, it is important to consider the extent to which the presence of training within the medical curricula of countries may be indicative of broader social and cultural infrastructure concerned with and understanding of CM, rather than a targeted intervention. Regardless, the attitudes present in our study suggest that these health care providers would be open to education and training to build awareness and understanding around CM and increase knowledge and adherence of best practices.

What our findings do communicate is an apparent gap between attitudes and current practices. Responses implied a limited understanding of protocols and laws. For example, slightly half of participants correctly indicated that the hospital does not have an existing care protocol for cases of CM and approximately one-third stated that they did not know the referral process for suspected cases of CM. Lack of training specific to CM and its symptoms may be partially responsible for this disconnect; however, the cultural context might have more of an influence. Globally, in high-income and LMIC alike, training to recognize and follow CM protocols in medical curriculums is minimal and only a small part of overall education [27, 31, 42]. For example, in the United States, only one state mandates that physicians complete the Identification and Reporting of Child Abuse and Maltreatment course before licensing [42]. Yet, poor knowledge of what constitutes CM and lack of best reporting practices are not observed uniformly across countries; studies from other countries have found baseline physician knowledge and reporting to be higher, although still limited in some respects [27, 29, 43, 44]. Health care providers in our sample are operating within a context where the harsh punishment of children is normalized; in the region, there has been found to be poor societal knowledge of what constitutes abuse and accepting attitudes towards such behaviors in community-based surveys [15]. Consequently, a major barrier to improving the capacity of health care providers to intervene is not a lack of concern or acceptance of CM, but rather the difficulty in training physicians to recognize normalized and accepted behaviors as in fact acts of CM within their community threshold. To accomplish this, awareness around definitions of CM and risk factors should be the focal point of training, rather than purely knowledge-based instruction around symptoms. These interventions in health care providers are needed jointly with administrative reforms in the hospital to strengthen coordination between the health system and the legal authorities. MGH where our study was based currently does not have a care protocol specific to CM, although health care providers in Mozambique are obligated by law to report suspected CM to the police [45].

### Limitations

The main limitations of this study are its small sample size of 60 health care providers and its use of purposive sampling. Given the study size and sampling methods, findings are not statistically generalizable to the greater population of health care providers in Mozambique. However, the study’s objectives were concerned with health care providers working in emergency services and studies of subpopulations of health care providers, such as pediatricians or family physicians, have utilized comparable sampling approaches [28, 35]. Another point of consideration stemming from the methodology of survey administration is the potential for social desirability bias. While the generally low levels of knowledge and adherence to best practices reported makes this unlikely, it is important to consider that positive attitudes may be overstated. Future surveys administered electronically or in other anonymizing manners would allow for comparisons and minimize the influence of social desirability bias.

Although the interpretation of our results is confined to one hospital, MGH is the largest hospital in the federal capital and serves almost half of the area’s population. As is common in LMICs, city hospitals are tasked with providing care to both urban and peri-urban populations and may be the only source of specialized care for those living in rural areas who may have to travel long distances [41, 46]. Therefore, studying urban hospitals in LMICs has the potential to yield significant benefits for the health system as a whole due to their large catchment populations and their tendency to possess greater resources for service delivery. The size and location of MGH make it one of the country’s most-equipped hospitals and inadequacies suggest that more research should also be focused on smaller and more rural hospitals in the country that likely experience increased deficiencies.

## Conclusion

Child maltreatment is a leading public health problem internationally. Health care providers in ECS play an important role in the prevention and management of cases of CM through appropriate services and referrals. This study found that ECS providers working at Mavalane General Hospital in Maputo, Mozambique, have limited knowledge of the forms of child maltreatment and their associated symptoms beyond physical abuse and most have not received training on the topic. Attitudes expressed by health care providers suggest a strong commitment to their role and openness to CM training. Thus, investing in programs to raise awareness to confront sociocultural attitudes and recognize warning signs integrated with education and training to build skills and confidence have the potential to strengthen response capacity to improve care for victims of CM in Mozambique and other LICs.

## Abbreviations

CM: Child maltreatment
ECS: Emergency care services
LIC: Low-income country
LMIC: Low- and middle-income country
MGH: Mavalane General Hospital
